# Incidence and risk factors of hepatocellular carcinoma in patients with hepatitis C in China and the United States

**DOI:** 10.1038/s41598-020-77515-y

**Published:** 2020-12-01

**Authors:** Ming Yang, Neehar D. Parikh, Huixin Liu, Elizabeth Wu, Huiying Rao, Bo Feng, Andy Lin, Lai Wei, Anna S. Lok

**Affiliations:** 1 Peking University People’s Hospital, Peking University Hepatology Institute, Peking University Health Science Center, Beijing, China; 2 Division of Gastroenterology and Hepatology, Department of Internal Medicine, University of Michigan, Ann Arbor, MI USA; 3 Department of Clinical Epidemiology and Biostatistics, Peking University People’s Hospital, Beijing, China; 4 The Molecular and Behavioral Neuroscience Institute, University of Michigan, Ann Arbor, MI USA

**Keywords:** Hepatitis C, Hepatocellular carcinoma

## Abstract

Hepatitis C virus (HCV) infection is the main cause of hepatocellular carcinoma (HCC) in the United States (US) and an increasingly common cause of HCC in China. We aimed to evaluate the incidence and risk factors of HCC in HCV patients in the US and China. 795 HCV RNA + patients without HCC from University of Michigan Health System (UMHS) in the US and 854 from Peking University Health Sciences Center (PUHSC) in China were prospectively followed for a median of 3.2 and 4.0 years, respectively. 45.4% UMHS and 16.2% PUHSC patients had cirrhosis. 57.6% UMHS and 52.0% PUHSC patients achieved SVR. 45 UMHS and 13 PUHSC patients developed HCC. Cumulative incidence of HCC at 5 years was 7.6% in UMHS and 1.8% in PUHSC cohort (*P* < 0.001). Ten patients not diagnosed with cirrhosis at enrollment but median APRI ≥ 2.0 developed HCC. Multivariate analysis showed age, gender, cirrhosis and APRI were predictors of HCC while study site and SVR were not. In this study of HCV patients, HCC incidence in the PUHSC cohort was lower than in the UMHS cohort, due to lower proportion of PUHSC patients with cirrhosis. APRI can identify risk of HCC among patients not diagnosed to have cirrhosis.

## Introduction

Globally, an estimated 71 million people have chronic hepatitis C virus (HCV) infection and approximately 399 000 people die from hepatitis C each year, primarily due to complications of cirrhosis and hepatocellular carcinoma (HCC)^1^. The prevalence of chronic HCV infection in the United States (US) is estimated to be 1.0–2.0%, affecting between 2.7–5.2 million people^2,3^. The prevalence of chronic HCV infection in China is estimated to be 1%, with approximately 10 million persons infected^4^. Complications related to chronic HCV infection usually arise 3–4 decades after infection^5^. The US is currently experiencing a peak in HCV-related complications due to the high incidence of HCV infection in the 1970s and 1980s, and chronic HCV infection is the leading cause of HCC in the US^6,7^. In China, the majority of patients with HCV were infected more recently than in the US, predominantly in the 1980s and 1990s. Due to the late introduction of and limited access to direct-acting antivirals (DAAs) in China, complications of HCV infection, including HCC, are expected to increase^8^.

Host (age, sex, genetics, obesity, diabetes, the presence of cirrhosis), virus (HCV genotype, coinfection with hepatitis B virus [HBV] or human immunodeficiency virus [HIV]), and environmental (alcohol, smoking, coffee) factors contribute to progression of hepatitis C^5,9^. Achievement of a sustained virologic response (SVR) after antiviral therapy is associated with a significant reduction in incidence of HCC; however, HCC risk is not eliminated even after SVR^10,11^.

In 2011, we initiated a parallel cohort study of patients with chronic HCV infection in the US and in China to compare the incidence and risk factors of clinical outcomes. We previously reported that at enrollment, a significantly higher proportion of US patients had cirrhosis (38.2% vs. 16.0%) and HCC (14.1% vs. 2.7%) than the Chinese patients^12^. The current study utilized prospective longitudinal data in these two cohorts to compare the incidence of HCC and to study factors associated with HCC development.

## Results

**Figure 1:**
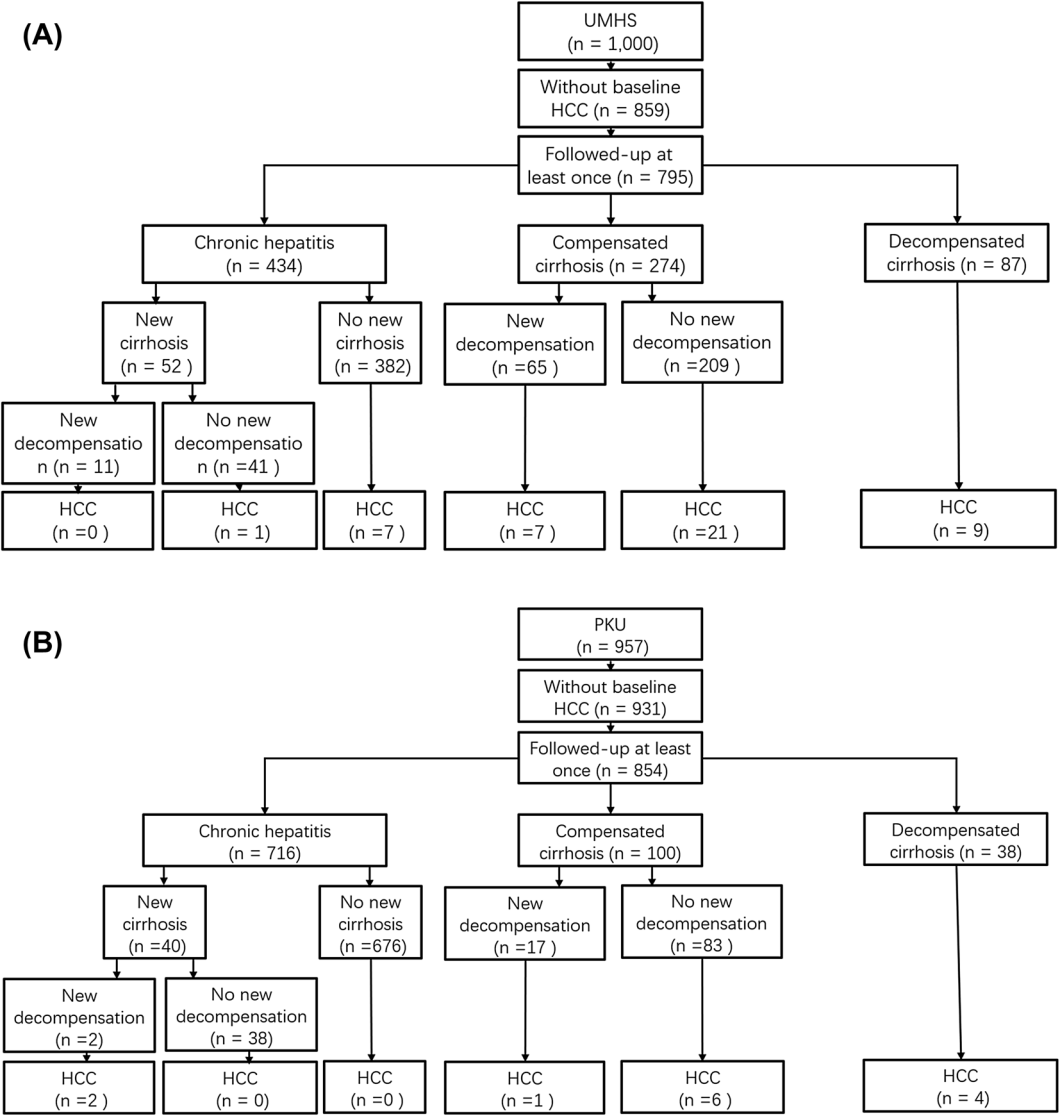
Study flow chart summarizing clinical outcomes and occurrence of HCC stratified by baseline liver disease stage, (**A**) UMHS cohort and (**B**) PUHSC cohort.

A total of 1957 patients were enrolled: 1000 at UMHS, and 957 at PUHSC. Of these, 167 were excluded for having HCC (141 UMHS and 26 PUHSC) at enrollment, and 141 (64 UMHS and 77 PUHSC) were excluded due to lack of follow-up. The remaining 1649 patients: 795 at UMHS and 854 at PUHSC were included in this analysis. Figure 1A,B show the outcomes of patients and occurrence of HCC stratified by baseline liver disease stage.

### Baseline characteristics of patients studied

UMHS patients were more likely to be men (57.4% vs. 48.2%), older (median age 57 vs. 53 years), obese (39.7% vs. 17.7%), and diabetic (21.3% vs. 9.5%) than PUHSC patients (Table 1). Compared to PUHSC patients, UMHS patients were more likely to be current/past drinkers (61.4% vs. 27.4%) or smokers (77.4% vs. 35.4%), and to consume coffee regularly (62.5% vs. 4.4%) but less likely to be anti-HBc positive (31.2% vs. 46.4%).

**Table 1:** Characteristics of patients in the UMHS and PUHSC cohorts.

	UMHS cohort (n = 795)	PUHSC cohort (n = 854)	*P* value
Age (years)	57 (52–60)	53 (47–59)	< 0.001
**Sex**			
Male	456 (57.4%)	412 (48.2%)	< 0.001
BMI*			< 0.001
Underweight and normal weight	199 (25.0%)	395 (46.2%)	
Overweight	280 (35.2%)	308 (36.1%)	
Obese	316 (39.7%)	151 (17.7%)	
**DM**			
Yes	169 (21.3%)	81 (9.5%)	< 0.001
**Alcohol**			< 0.001
Never	307 (38.6%)	620 (72.6%)	
Current/past use	488 (61.4%)	234 (27.4%)	
**Smoking**			< 0.001
Never	180 (22.6%)	552 (64.6%)	
Current/past use	615 (77.4%)	302 (35.4%)	
**Coffee**			< 0.001
Never	298 (37.5%)	816 (95.5%)	
Current/past consumption	497 (62.5%)	38 (4.4%)	
**HCV genotype**			< 0.001
Non-type I	127 (16.2%)	239 (28.5)	
Type I	655 (83.8%)	598 (71.4)	
**Anti-HBc**			< 0.001
Negative	544 (68.8%)	458 (53.6%)	
Positive	247 (31.2%)	396 (46.4%)	
**Albumin (g/dl)**			< 0.0001
< 3.0	64 (8.1%)	11 (1.3%)	
> = 3.0	730 (91.9%)	836 (98.7%)	
**Total bilirubin (mg/dl)**			< 0.0001
< 2.0	714 (89.9%)	812 (96.0%)	
> = 2.0	80 (10.1%)	34 (4.0%)	
ALT (U/L)	60.0 (41.0, 94.0)	43.0 (28.0, 68.0)	< 0.0001
AST (U/L)	60.0 (41.0, 95.0)	40.0 (28.0, 62.0)	< 0.0001
Creatinine (mg/dL)	0.8 (0.7, 1.0)	0.7 (0.6, 0.8)	< 0.0001
**INR**			< 0.0001
< 1.2	548(73.0%)	723 (96.8%)	
> = 1.2	203 (27.0%)	24 (3.2%)	
**Platelet (1000/μl)**			< 0.0001
< 100	211 (26.5%)	131 (15.5%)	
> = 100	584 (73.5%)	712 (84.5%)	
**Baseline APRI**			< 0.001
< = 1.0	386 (48.5%)	596 (70.7%)	
> 1.0- < = 2.0	161 (20.3%)	130 (15.4%)	
> 2.0	248 (31.2%)	117 (13.9%)	
**Baseline FIB-4**			< 0.001
< 1.45	172 (21.6%)	275 (32.7%)	
1.45–3.25	260 (32.7%)	361 (42.9%)	
> 3.25	363 (45.7%)	206 (24.4%)	
**Liver disease stage**			< 0.001
No cirrhosis	434 (54.6%)	716 (83.8%)	
Cirrhosis	361 (45.4%)	138 (16.2%)	
**SVR**			0.02
Never achieved	337 (42.4%)	410 (48.0%)	
Achieved	458 (57.6%)	444 (52.0%)	

Data presented as median (IQR) for continuous variables or number (percent) for categorical variables.

*BMI* body mass index, *DM* diabetes mellitus, *HCV* hepatitis C virus, *Anti-HBc* antibody to hepatitis B core antigen, *APRI* aspartate aminotransferase to platelet ratio index, *FIB-4* Fibrosis index based on 4 factors, *SVR* sustained virologic response.

*BMI: for the UMHS cohort, underweight and normal weight: BMI < 25, overweight: 25 ≤ BMI < 30, obese: BMI ≥ 30; for the PUHSC cohort, underweight and normal weight: BMI < 24, overweight: 24 ≤ BMI < 28, obese: BMI ≥ 28.

The UMHS cohort included 434 (54.6%) patients without cirrhosis and 361 (45.4%) with cirrhosis of whom 87 had history of decompensation; while the PUHSC cohort included 716 (83.8%) patients without cirrhosis and 138 (16.2%) with cirrhosis of whom 38 had history of decompensation (Table 1). Patients with cirrhosis had significantly higher aspartate aminotransferase to platelet ratio index (APRI)^13^ (median 2.2 vs. 0.6) and higher Fibrosis index based on 4 factors (FIB-4)^14^ (median 6.4 vs. 1.7) than those with no cirrhosis.

Non-HCC outcomes were previously reported by our team^15^ and summarized in Fig. 1A,B. In brief, 76 patients in UMHS cohort (65 with and 11 without baseline cirrhosis) and 19 PUHSC patients (17 with and 2 without baseline cirrhosis) experienced new decompensation, 17 (16 UMHS and 1 PUHSC) patients underwent liver transplantation, and 66 (43 UMHS and 23 PUHSC patients) died from liver disease during the study.

### Incidence of HCC

During a total follow-up of 5850.7 person-years (2519.0 for UMHS cohort and 3331.7 for PUHSC cohort), 45 UMHS patients and 13 PUHSC patients were diagnosed with HCC. Of these, 37 (9 decompensated) UMHS and 11 (4 decompensated) PUHSC patients had cirrhosis at enrollment while 8 UMHS and 2 PUHSC patients did not meet criteria for cirrhosis at enrollment. Diagnosis of HCC was biopsy proven in 8 UMHS patients and 2 PUHSC patients, and based on imaging in the remainder.

**Figure 2:**
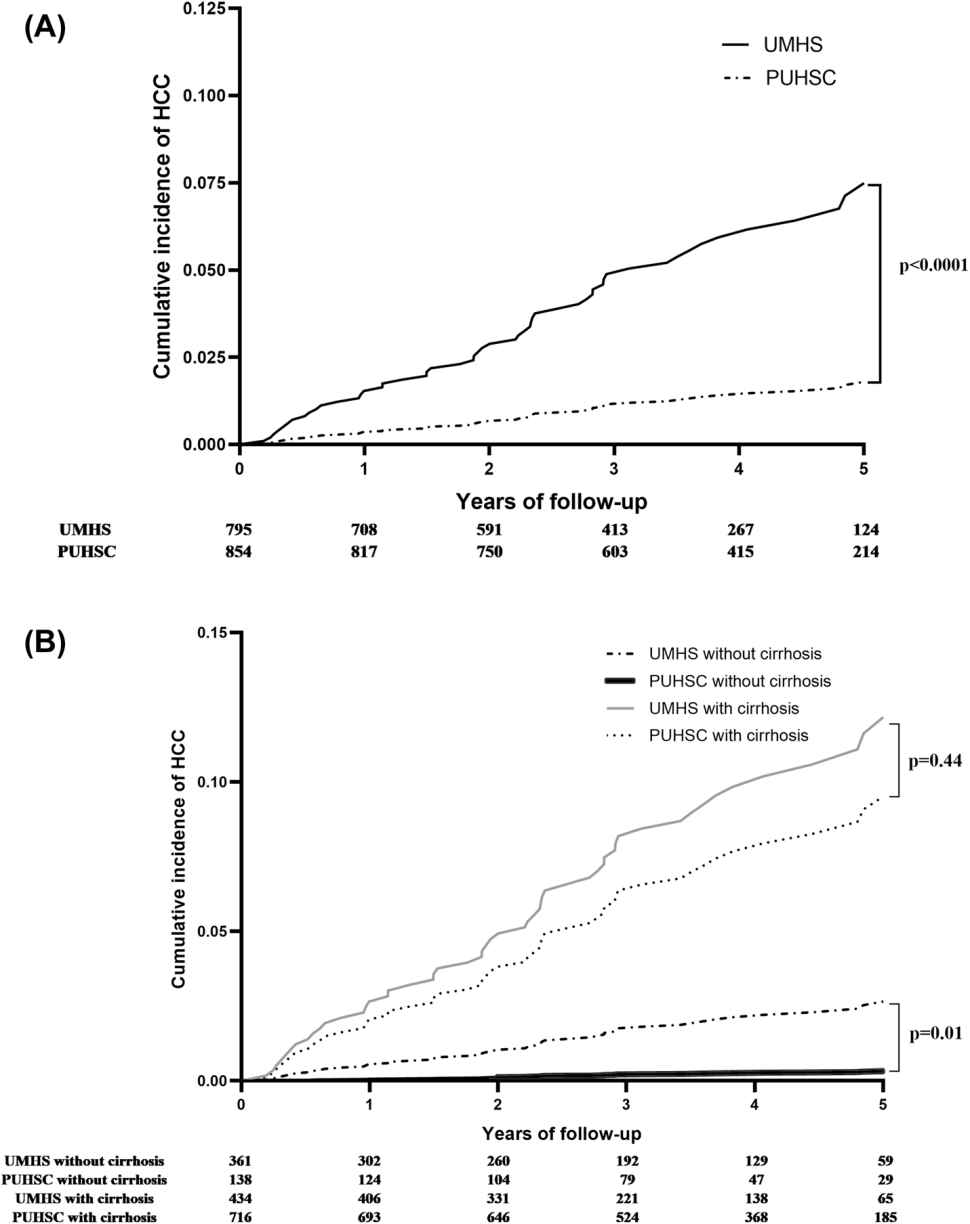
Cumulative incidence of HCC among UMHS and PUHSC patients, (**A**) all patients and (**B**) stratified for cirrhosis at enrollment.

The incidence of HCC was 1.8 (95% CI 1.3–2.4) per 100 person-years in the UMHS cohort and 0.4 (95% CI 0.2–0.7) per 100 person-years in the PUHSC cohort (P < 0.001). There were no differences in incidence of HCC among the three Chinese sites: 0.7 (95% CI 0.3–1.3), 0.2 (95% CI 0.1–0.6), and 0.2 (95% CI 0.03–1.5) per 100 person-years in Beijing, Gu’an, and Kuangcheng, respectively. The cumulative incidence of HCC at 1, 3, and 5 years of follow-up was 1.5%, 4.9% and 7.6%, respectively in the UMHS cohort, and 0.4%, 1.2% and 1.8%, respectively in the PUHSC cohort (P < 0.001) (Fig. 2A).

When patients were stratified by cirrhosis status at enrollment, the incidence of HCC in the UMHS cohort was significantly higher than in the PUHSC cohort among patients without cirrhosis: 0.6 (95% CI 0.3–1.2) vs. 0.1 (95% CI 0.02–0.3) per 100 person-years (P = 0.01) and cumulative incidence at 5 years 2.9% (95% CI 1.5–5.4%) vs. 0.4% (95% CI 0.1–2.0%), but not in those with cirrhosis: 3.3 (95% CI 2.4–4.5) vs. 2.4 (95% CI 1.3–4.3) per 100 person-years (P = 0.44) and cumulative incidence at 5 years: 15.2% (95% CI 11.2–20.8%) vs. 10.9% (95% CI 5.9–20.0%) (Fig. 2B). When patients with cirrhosis were further stratified, the incidence of HCC in those with and without decompensation was also similar in the two cohorts: 5.4 (95% CI 2.7–10.3) vs. 3.7 (95% CI 1.4–10.0) per 100 person-years, and 2.9 (95% CI 2.0–4.2) vs. 2.0 (95% CI 1.0–4.2) per 100 person-years, respectively.

### SVR and HCC

During follow-up, 516 UMHS and 505 PUHSC patients received HCV treatment. Of these, 456 UMHS and 214 PUHSC patients received interferon-free DAA treatment. SVR was achieved in 458 (57.6% overall and 88.0% of those treated) UMHS patients and 444 (52.0% overall and 86.7% of those treated) PUHSC patients.

Among patients without baseline cirrhosis, 10 patients (8 UMHS and 2 PUHSC) developed HCC before achieving SVR, while no patients developed HCC after a total of 907 person-year post-SVR follow-up (Table 2). Among patients with baseline cirrhosis, 40 patients (30 UMHS and 10 PUHSC) developed HCC before achieving SVR, while 8 patients (7 UMHS and 1 PUHSC) developed HCC after achieving SVR (Table 2).

**Table 2:** Incidence of HCC in UMHS and PUHSC cohorts before and after SVR (SVR as a time-dependent variable).

	UMHS cohort			PUHSC cohort			Total			HR (95% CI)	*P* value
	Patients at risk	Patients developed HCC	HCC incidence (per 100 person-year)	Patients at risk	Patients developed HCC	HCC incidence (per 100 person-year)	Patients at risk	Patients developed HCC	HCC incidence (per 100 person-year)		
**No cirrhosis group**											
Before SVR	434	8	0.7 (0.3–1.4)	716	2	0.1 (0.02–0.3)	1150	10	0.3 (0.1–0.5)		
After SVR	294	0	NA	385	0	NA	679	0	NA	NA	
Total	434	8	0.6 (0.3–1.1)	716	2	0.1 (0.02–0.3)	1150	10	0.2 (0.1–0.4)		
**Cirrhosis group**											
Before SVR	361	30	3.4 (2.4–4.9)	138	10	2.5 (1.3–4.7)	499	40	3.1 (2.3–4.3)	1.0	
After SVR	164	7	2.9 (1.4–6.0)	59	1	1.6 (0.2–11.5)	223	8	2.6 (1.3–5.2)	0.9 (0.4–1.9)	0.74
Total	361	37	3.3 (2.4–4.5)	138	11	2.4 (1.3–4.3)	499	48	3.0 (2.3–4.0)		
**Overall**											
Before SVR	795	38	1.9 (1.4–2.6)	854	12	0.4 (0.2–0.7)	1649	50	1.0 (0.8–1.3)	1.0	
After SVR	458	7	1.4 (0. 7–2.9)	444	1	0.2 (0.03–1.6)	902	8	0.8 (0.4–1.7)	0.8 (0.4–1.6)	0.53
Total	795	45	1.8 (1.3–2.4)	854	13	0.4 (0.2–0.7)	1649	58	1.0 (0.8–1.3)		

*HCC* Hepatocellular carcinoma, *SVR* sustained virologic response, *HR* hazard ratio, *NA* not applicable.

To further examine the impact of SVR on HCC incidence, we combined the two cohorts and stratified the patients by cirrhosis status at baseline and analyzed SVR as a time-dependent variable (Table 2). The incidence of HCC was 2.6 vs. 3.1 per 100 person-years (HR 0.9, 95% CI 0.4–1.9) in cirrhosis patients with vs. those without SVR; and 0 vs. 0.3 per 100 person-years in non-cirrhosis patients with vs. those without SVR. When SVR was analyzed as time-fixed variable (Supplementary Table 1), incidence of HCC was 0.8 vs. 6.2 per 100 person-years (HR 0.2, 95% CI 0.1–0.4) in cirrhosis patients with vs. those without SVR; and 0 vs. 0.6 per 100 person-years in non-cirrhosis patients with vs. those without SVR.

### Factors associated with HCC development

In both cohorts, patients with HCC development were significantly older, more likely to have cirrhosis and had higher APRI and FIB-4 at enrollment and less likely to have achieved SVR during follow-up. However, there were no differences in BMI, diabetes, use of alcohol, tobacco or coffee, HCV genotype and prevalence of anti-HBc compared to those without HCC (Table 3). Results of univariate competing risk analysis are shown in Supplementary Table 2.

**Table 3:** Baseline characteristics of patients with and without HCC development.

	UMHS no HCC	UMHS with HCC	*P* value	PUHSC no HCC	PUHSC with HCC	*P* value
No	750	45		841	13	
**Demographics**						
Sex, male	425 (56.7%)	31 (68.9%)	0.107	403 (47.9%)	9 (69.2%)	0.13
Age, years	56 (52–60)	61 (59–64)	< 0.001	53 (47–59)	58 (55–61)	0.005
**Metabolic factors**						
**BMI**			0.407			0.46
Underweight and normal weight	189 (25.2%)	10 (22.2%)		390 (46.4%)	5 (38.5%)	
Overweight	260 (34.7%)	20 (44.4%)		304 (36.1%)	4 (30.8%)	
Obese	301 (40.1%)	15 (33.3%)		147 (17.5%)	4 (30.8%)	
DM	155 (20.7%)	14 (31.1%)	0.096	80 (9.5%)	1 (7.7%)	1
**Environmental factors**						
Alcohol: current or past	463 (61.7%)	25 (55.6%)	0.408	228 (27.1%)	6 (46.2%)	0.21
Smoking: current or past	579 (77.2%)	36 (80.0%)	0.663	295 (35.1%)	7 (53.8%)	0.24
Coffee: current or past	467 (62.3%)	30 (66.7%)	0.554	37 (4.4%)	1 (7.7%)	0.45
**Viral factors**						
HCV genotype	N = 739	N = 43	0.994	N = 824	N = 13	0.12
Genotype 1	619 (83.8%)	36 (83.7%)		586 (71.1%)	12 (92.3%)	
Anti-HBc	N = 746	N = 45	0.753	N = 841	N = 13	0.59
Positive	232 (31.1%)	15 (33.3%)		389 (46.3%)	7 (53.8%)	
**Liver disease severity**						
APRI	N = 750	N = 45	< 0.001	N = 830	N = 13	< 0.001
< = 0.5	190 (25.3%)	0 (0.0%)		345 (41.6%)	0 (0.0%)	
> 0.5–< = 1.0	191 (25.5%)	5 (11.1%)		251 (30.2%)	0 (0.0%)	
> 1.0–< = 2.0	149 (19.9%)	12 (26.7%)		127 (15.3%)	3 (23.1%)	
> 2.0	220 (29.3%)	28 (62.2%)		107 (12.9%)	10 (76.9%)	
FIB-4	N = 750	N = 45	< 0.001	N = 829	N = 13	< 0.001
< 1.45	172 (22.9%)	0 (0.0%)		275 (33.2%)	0 (0.0%)	
1.45–3.25	256 (34.1%)	4 (8.9%)		360 (43.4%)	1 (7.7%)	
> 3.25	322 (42.9%)	41 (91.1%)		194 (23.4%)	12 (92.3%)	
**Disease group at enrollment**			< 0.001			< 0.001
No cirrhosis	426 (56.8%)	8 (17.8%)		714 (84.9%)	2 (15.4%)	
Cirrhosis	324 (43.2%)	37 (82.2%)		127 (15.1%)	11 (84.6%)	
SVR^§^	451 (60.1%)	7 (15.6%)	< 0.001	443 (52.7%)	1 (7.7%)	0.001

Data presented as median (IQR) for continuous variables or number (percent) for categorical variables.

*HCC* Hepatocellular carcinoma, *BMI* body mass index, *DM* diabetes mellitus, *HCV* hepatitis C virus, *Anti-HBc* antibody to hepatitis B core antigen, *APRI* aspartate aminotransferase to platelet ratio index, *FIB-4* Fibrosis index based on 4 factors, *SVR* sustained virologic response.

^§^SVR here was a time-fixed variable.

**Figure 3:**
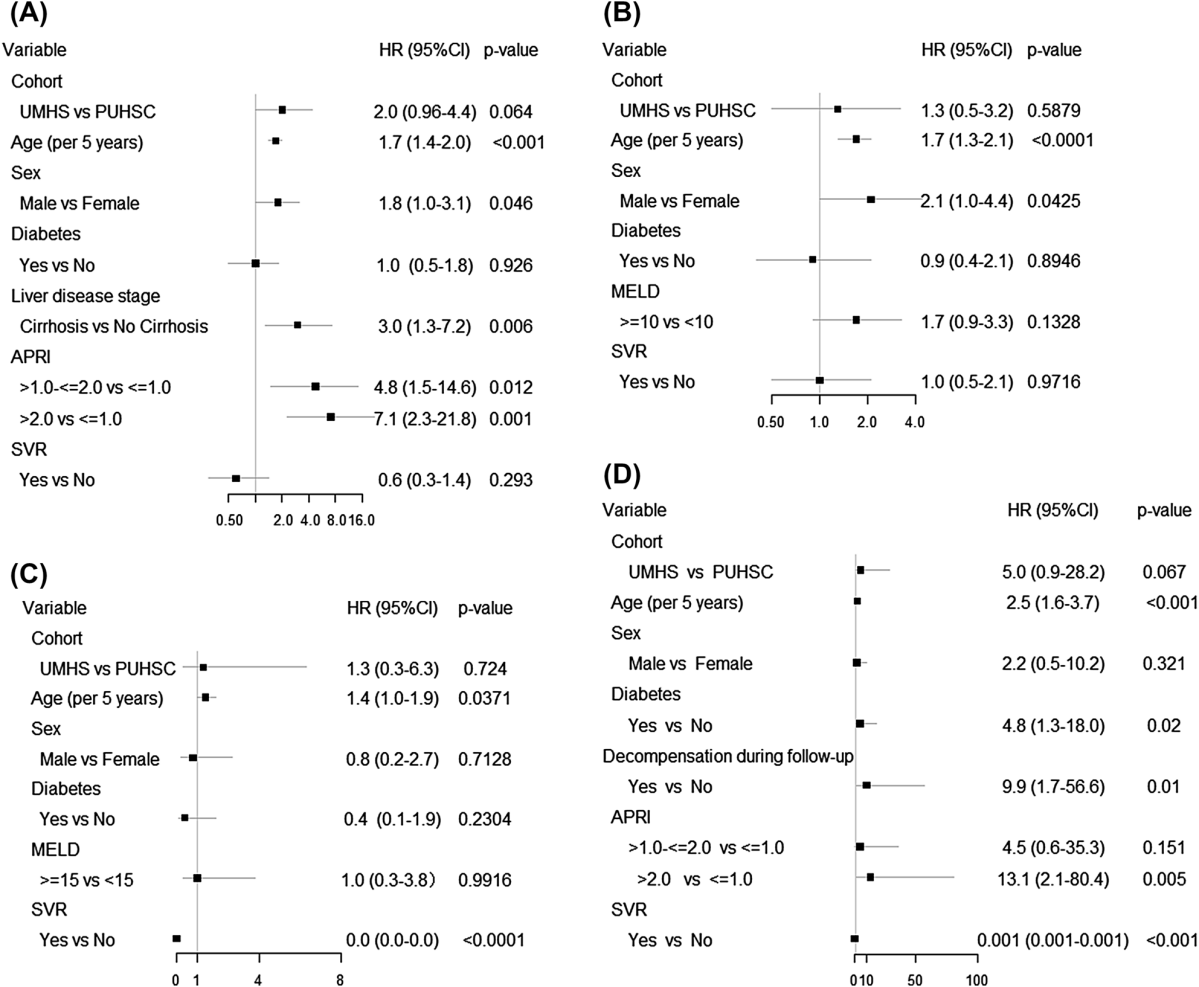
Risk factors for HCC in the combined UMHS and PUHSC cohorts, (**A**) all patients, (**B**) patients with compensated cirrhosis, (**C**) patients with decompensated cirrhosis, and (**D**) patients with no cirrhosis.

Multivariate Cox regression analysis showed that older age, male sex, higher APRI, and baseline cirrhosis were associated with a higher risk of HCC in the combined cohort, while study site, diabetes and SVR were not (Fig. 3A). Analysis of risk factors for HCC identified older age and male sex but not SVR in the subgroup with compensated cirrhosis (Fig. 3B) and older age, and SVR in the subgroup with decompensated cirrhosis (Fig. 3C). When the two cohorts were separately analyzed, older age, higher APRI and baseline cirrhosis were associated with a higher risk of HCC in the UMHS cohort (Supplementary Fig. 1), while no risk factor of HCC was found in the PUHSC cohort (Supplementary Fig. 2).

### HCC in non-cirrhosis patients

Ten patients (8 UMHS and 2 PUHSC) not diagnosed to have cirrhosis at enrollment developed HCC during follow-up. Median APRI at baseline was 2.0 and FIB-4 was 4.5 in these 10 patients, compared to 0.6 and 1.7, respectively in those who did not develop HCC. Of note, none of 509 patients with baseline APRI ≤ 0.5 and none of 437 patients with baseline FIB-4 < 1.45 developed HCC during follow-up, while 5 of 86 (5.8%) patients with baseline APRI > 2.0 and 8 of 161 (5.0%) with baseline FIB-4 > 3.25 developed HCC. Of the 10 patients who developed HCC, 3 met protocol criteria for cirrhosis at the time of HCC diagnosis, and an additional 3 had APRI > 2.0 and FIB-4 > 3.25 but did not meet protocol criteria for cirrhosis at the time of HCC diagnosis.

Due to the small number of HCC outcome, analysis of risk factors of HCC in the group with no cirrhosis at baseline is exploratory. Multivariate analysis showed that older age, diabetes, APRI > 2.0, and decompensation during follow-up were independently associated with a higher risk of HCC, while achieving SVR was independently associated with a lower risk of HCC in the combined cohort of UMHS and PUHSC patients with no cirrhosis at baseline (Fig. 3D).

## Discussion

Chronic HCV infection is the leading cause of HCC in the US and an increasingly common cause of HCC in China. Our study sought to compare the incidence of HCC in UMHS and PUHSC HCV cohorts and to identify the risk factors of HCC development. We found that HCC incidence in the PUHSC cohort was lower than in the UMHS cohort, mainly due to a lower proportion of PUHSC patients with cirrhosis.

Other differences in the two cohorts may also contribute to the higher incidence of HCC in the UMHS cohort, e.g., more men, higher BMI, more diabetes and more patients with history of regular alcohol or tobacco use. Male sex had been shown in many studies to be a predictor of HCC^16^, which was confirmed in the combined cohort and UMHS cohort. Previous studies demonstrated that obesity, diabetes, alcohol or tobacco use increased risk of HCC in HCV patients^17–20^. In our study, diabetes and tobacco use were associated with a higher risk of HCC on univariate analysis but not on multivariate analysis in the combined cohort. We were not able to confirm the associations of higher BMI and alcohol use with HCC possibly due to the small number of patients with HCC.

Twenty patients in the PUHSC cohort and none in the UMHS cohort were HBsAg positive but none of them developed HCC during follow-up. There was a lower prevalence of anti-HBc and more frequent coffee consumption in the UMHS cohort. We found no difference in prevalence of anti-HBc between patients with and those without HCC development in the combined cohort and also when the two cohorts were separately analyzed. Reports on the role of anti-HBc positivity and occult HBV infection in HCC development among patients with chronic HCV infection showed conflicting results^21,22^. Coffee consumption had been demonstrated to have a protective effect against HCC^23^. In our study, coffee consumption was associated with lower HCC incidence on univariate analysis but not on multivariate analysis in the combined cohort.

In our study, the most consistent predictor of HCC was cirrhosis. The higher proportion of UMHS patients with cirrhosis at enrollment resulted in a significantly higher incidence of HCC in the UMHS than the PUHSC cohort. When patients were stratified for cirrhosis at enrollment, there was no difference in incidence of HCC in the two cohorts among the patients with cirrhosis, overall and when stratified for presence or absence of decompensation. Among the patients with no cirrhosis, a higher incidence of HCC in the UMHS cohort persisted likely because the UMHS patients had more advanced fibrosis when compared to the PUHSC cohort (median APRI 0.62 vs. 0.53). Indeed, of the 10 patients with no cirrhosis at baseline, in whom HCC developed during follow-up, 3 met protocol criteria for cirrhosis and an additional 3 had APRI > 2.0 and FIB-4 > 3.25 at the time of HCC diagnosis, and 4 of these 6 were UMHS patients. While most HCV-related HCC develops in patients with cirrhosis, it can occur in those with advanced fibrosis^24^. We found that APRI and FIB-4 can stratify the risk of HCC in patients who did not have a diagnosis of cirrhosis. Among the patients with no cirrhosis at enrollment, none of the patients with baseline APRI ≤ 0.5 or FIB-4 < 1.45 developed HCC during follow-up. Validation of our finding will help to identify HCV patients with advanced fibrosis or undiagnosed cirrhosis who might benefit from HCC surveillance.

Most studies showing a significant reduction in incidence of HCC after achieving SVR have used SVR as a time-fixed variable^10,11,25^. The results of these studies can be potentially biased even when stratified for cirrhosis status at enrollment because patients with more advanced liver disease are more likely to develop HCC and at the same time less likely to achieve SVR. In a French cohort study of 1270 HCV-associated cirrhosis patients (336 treated with DAAs and 934 treated with interferon) in which SVR was used as a time-dependent variable, SVR was independently associated with a lower risk of HCC (HR 0.44; 0.29–0.69)^26^.

In our study, we used SVR as a time-fixed variable as well as a time-dependent variable. Using SVR as a time-fixed variable, we found that SVR was associated with a significant reduction in incidence of HCC. When we used SVR as a time-dependent variable, SVR was not associated with a statistically significant reduction in incidence of HCC overall, possibly because of the short duration of post-SVR follow-up, median (IQR) 12.0 (4.6–19.4) months in UMHS cohort and 0 (0–22.5) months in PUHSC cohort. However, SVR was associated with a statistically significant reduction in incidence of HCC in patients with no cirrhosis and those with decompensated cirrhosis at baseline. We also found that the incidence of HCC after SVR was still high in cirrhosis patients while no HCC occurred after SVR in non-cirrhosis patients. These data highlight the importance of continued HCC surveillance in patients with cirrhosis prior to achieving SVR, and the need to diagnose and treat patients early, prior to cirrhosis.

Our study has notable strengths. The parallel study design and the prospective nature of our study allow us to compare the rate of incident HCC in two cohorts of patients with chronic HCV infection in the US and in China. The use of standardized protocol, manual of procedures, and criteria for diagnosis of cirrhosis and HCC, and source documents to verify these diagnoses provide confidence in our results. There are several limitations in our study. First, while this study enrolled more than 1600 patients, the number is too small to be representative of patients with chronic HCV infection in the US and in China. Second, our follow-up duration, in particular the duration of post-SVR follow-up, was short, limiting our ability to study the impact of SVR on long-term risk of HCC. Third, the number of HCC events was small limiting the power to detect risk factors within as well as between cohorts. Fourth, there was ascertainment bias in our study because if a patient was lost to follow-up and developed HCC then we would have no way to record this HCC event.

In summary, our study found that HCC incidence in the PUHSC cohort was significantly lower than in the UMHS cohort. This difference is mainly due to less advanced liver disease in the PUHSC cohort. Incidence of HCC was decreased but not eliminated in cirrhosis patients who achieved SVR. By contrast, no HCC was observed in non-cirrhosis patients who achieved SVR. Among patients with no cirrhosis, high APRI identified those who were at risk of HCC. Our data support that patients with chronic HCV infection should be treated prior to development of cirrhosis to prevent liver-related complications, including HCC.

## Methods

### Study design and patients

We conducted a prospective study of two parallel cohorts of patients with chronic HCV infection recruited in Ann Arbor, US (University of Michigan Health System, UMHS) and in Beijing, China (Peking University Health Sciences Center, PUHSC). Patients in China were enrolled from three sites: Peking University People’s Hospital in Beijing, and Gu’an and Kuancheng clinics in Hebei. A description of the study design had been previously reported^12^. In brief, consecutive adults age ≥ 18 years with chronic HCV infection (HCV RNA positive) were enrolled between September 2011 and July 2015. Patients who had undergone liver transplantation, known coinfection with HIV, life expectancy < 12 months due to extra-hepatic illnesses, or were receiving HCV treatment at enrollment, were excluded. Patients enrolled at both centers were evaluated using an identical protocol. All patients except those with HCC at enrollment were prospectively followed. Patients with cirrhosis were evaluated every 6 months while those without cirrhosis were evaluated every 12 months. Follow-up continued until patient died, developed HCC, received liver transplantation, withdrew informed consent, or when the study was closed in December 2017. Patients who did not have any follow-up visit after enrollment were excluded from this analysis. The research protocol was approved by the Institutional Review Boards of both the University of Michigan and Peking University Health Science Center and conducted in accordance with the Declaration of Helsinki. Written informed consent was obtained from all subjects.

Protocol, surveys, and data forms were developed in English and then translated into Chinese, and data were entered into a web-based database.

### Clinical parameters

Demographic (race/ethnicity, age, sex), clinical (medical history, current medications, and family history of liver disease and HCC), and laboratory data (blood counts, liver panel, creatinine, international normalized ratio [INR], alpha fetoprotein, HCV genotype, HCV RNA, hepatitis B surface antigen [HBsAg], antibody to hepatitis B core antigen [anti-HBc]), abdominal imaging (ultrasound, computed tomography [CT], magnetic resonance imaging [MRI]), liver elastography and liver histology results were collected at baseline and at each follow-up visit.

Alcohol, tobacco and coffee consumption were assessed using a standardized questionnaire. Regular alcohol use was defined as at least 1 drink/day, regular tobacco use was at least one cigarette/day, and regular coffee consumption at least 1 cup/day.

For American patients, overweight was defined as body mass index (BMI) ≥ 25 but < 30, and obesity as BMI ≥ 30 kg/m^2^ ^27^. For Chinese patients, overweight was defined as BMI ≥ 24 but < 28, and obesity as BMI ≥ 28 kg/m^2^ ^28^. Diabetes mellitus (DM) was defined by medical history or use of medications for treatment of diabetes; and for those with no history of diabetes by fasting blood glucose ≥ 126 mg/dL or random blood glucose ≥ 200 mg/dL.

### Assessment of liver disease

Diagnosis of cirrhosis was based on histology when available. In the absence of histology, diagnosis of cirrhosis was based on evidence of clinical decompensation or 2 of the following 4 criteria: radiological imaging showing features of cirrhosis (nodular liver, varices or splenomegaly), platelet count < 100 × 10^9^/L in the absence of other explanations, liver stiffness measurement by vibration controlled transient elastography (Fibroscan) > 13 kPa, and gastro-esophageal varices on endoscopy. HCC was diagnosed by histology or by triple phase CT or MRI per the American Association for the Study of Liver Diseases guidelines^29^. Source documents supporting the diagnosis of cirrhosis and HCC were collected and investigators from each country audited the documents from the other country to confirm these diagnoses.

### Statistical analyses

Data were analyzed using SAS 9.4 (SAS Institute, Cary, NC). Categorical data are presented as number (percent) and continuous data as median (interquartile range, IQR). T-test or Mann–Whitney test was used to compare continuous variables according to the data distribution and chi-square test for comparison of categorical data. Kaplan–Meier analysis was used to estimate cumulative incidence of HCC. Univariate and multivariate competing risk models were used to identify risk factors of HCC. Death or liver transplantation before HCC diagnosis was considered as competing risk event, and SVR was included as a time-dependent variable. Due to the short duration of post-SVR follow-up, analysis was also performed with SVR as time-fixed variable. Demographic, clinical and environmental factors with P < 0.1 on univariate analysis were incorporated in the full model. P values < 0.05 were considered statistically significant.

## Supplementary information

Supplementary Figure 1.

Supplementary Figure 2.

Supplementary Information 3.

## References

[1] World Health Organization. Hepatitis C. Available at: http://www.who.int/news-room/fact-sheets/detail/hepatitis-c. Updated July 2020.

[2] Denniston MM (2014). Chronic hepatitis C virus infection in the United States, National Health and Nutrition Examination Survey 2003 to 2010. Ann. Intern. Med..

[3] Chak E, Talal AH, Sherman KE, Schiff ER, Saab S (2011). Hepatitis C virus infection in USA: An estimate of true prevalence. Liver Int..

[4] Wei L, Hou JL (2015). The guideline of prevention and treatment for hepatitis C: A 2015 update. Zhonghua ganzangbing zazhi.

[5] Westbrook RH, Dusheiko G (2014). Natural history of hepatitis C. J. Hepatol..

[6] Sayiner M (2016). Presence of hepatitis C (HCV) infection in Baby Boomers with Medicare is independently associated with mortality and resource utilisation. Aliment. Pharmacol. Ther..

[7] de Martel C, Maucort-Boulch D, Plummer M, Franceschi S (2015). World-wide relative contribution of hepatitis B and C viruses in hepatocellular carcinoma. Hepatology (Baltimore, MD).

[8] Qin Q (2015). Hepatitis C virus infection in China: An emerging public health issue. J. Viral. Hepat..

[9] El-Serag HB (2012). Epidemiology of viral hepatitis and hepatocellular carcinoma. Gastroenterology.

[10] El-Serag HB, Kanwal F, Richardson P, Kramer J (2016). Risk of hepatocellular carcinoma after sustained virological response in Veterans with hepatitis C virus infection. Hepatology (Baltimore, MD).

[11] Kanwal F (2017). Risk of hepatocellular cancer in HCV patients treated with direct-acting antiviral agents. Gastroenterology.

[12] Rao H (2017). The higher prevalence of truncal obesity and diabetes in American than Chinese patients with chronic hepatitis C might contribute to more rapid progression to advanced liver disease. Aliment. Pharmacol. Ther..

[13] Wai CT (2003). A simple noninvasive index can predict both significant fibrosis and cirrhosis in patients with chronic hepatitis C. Hepatology (Baltimore, MD).

[14] Sterling RK (2006). Development of a simple noninvasive index to predict significant fibrosis in patients with HIV/HCV coinfection. Hepatology (Baltimore, MD).

[15] Rao H (2020). Comparison of clinical outcomes and impact of SVR in American and Chinese patients with chronic hepatitis C. JHEP Rep..

[16] White DL (2012). Higher serum testosterone is associated with increased risk of advanced hepatitis C-related liver disease in males. Hepatology (Baltimore, MD).

[17] Arnold M (2016). Obesity and cancer: An update of the global impact. Cancer Epidemiol..

[18] Huang YW (2015). Increased risk of hepatocellular carcinoma in chronic hepatitis C patients with new onset diabetes: A nation-wide cohort study. Aliment. Pharmacol. Ther..

[19] Hassan MM (2002). Risk factors for hepatocellular carcinoma: Synergism of alcohol with viral hepatitis and diabetes mellitus. Hepatology (Baltimore, MD).

[20] Yu MC, Yuan JM (2004). Environmental factors and risk for hepatocellular carcinoma. Gastroenterology.

[21] Ikeda K (2007). Antibody to hepatitis B core antigen and risk for hepatitis C-related hepatocellular carcinoma: A prospective study. Ann. Intern. Med..

[22] Lok AS (2011). Occult and previous hepatitis B virus infection are not associated with hepatocellular carcinoma in United States patients with chronic hepatitis C. Hepatology (Baltimore, MD).

[23] Bravi F, Bosetti C, Tavani A, Gallus S, La Vecchia C (2013). Coffee reduces risk for hepatocellular carcinoma: An updated meta-analysis. Clin. Gastroenterol. H..

[24] Lok AS (2011). Maintenance peginterferon therapy and other factors associated with hepatocellular carcinoma in patients with advanced hepatitis C. Gastroenterology.

[25] Ioannou GN, Green PK, Berry K (2017). HCV eradication induced by direct-acting antiviral agents reduces the risk of hepatocellular carcinoma. J. Hepatol..

[26] Nahon P (2018). Incidence of hepatocellular carcinoma after direct antiviral therapy for HCV in patients with cirrhosis included in surveillance programs. Gastroenterology.

[27] Obesity: preventing and managing the global epidemic. Report of a WHO consultation. *World Health Organization technical report series* **894**, 1–253 (2000). 11234459

[28] Zhou B (2002). Predictive values of body mass index and waist circumference to risk factors of related diseases in Chinese adult population. Zhonghua liuxingbingxue zazhi.

[29] Marrero JA (2018). Diagnosis, Staging, and Management of Hepatocellular Carcinoma: 2018 Practice Guidance by the American Association for the Study of Liver Diseases. Hepatology (Baltimore, MD).

