# Supplementary figures and images for: Incidence and risk factors of hepatocellular carcinoma in patients with hepatitis C in China and the United States
Source: Sci Rep. 2020 Dec 1;10:20922. doi: 10.1038/s41598-020-77515-y (PMC7708980; doi:10.1038/s41598-020-77515-y)

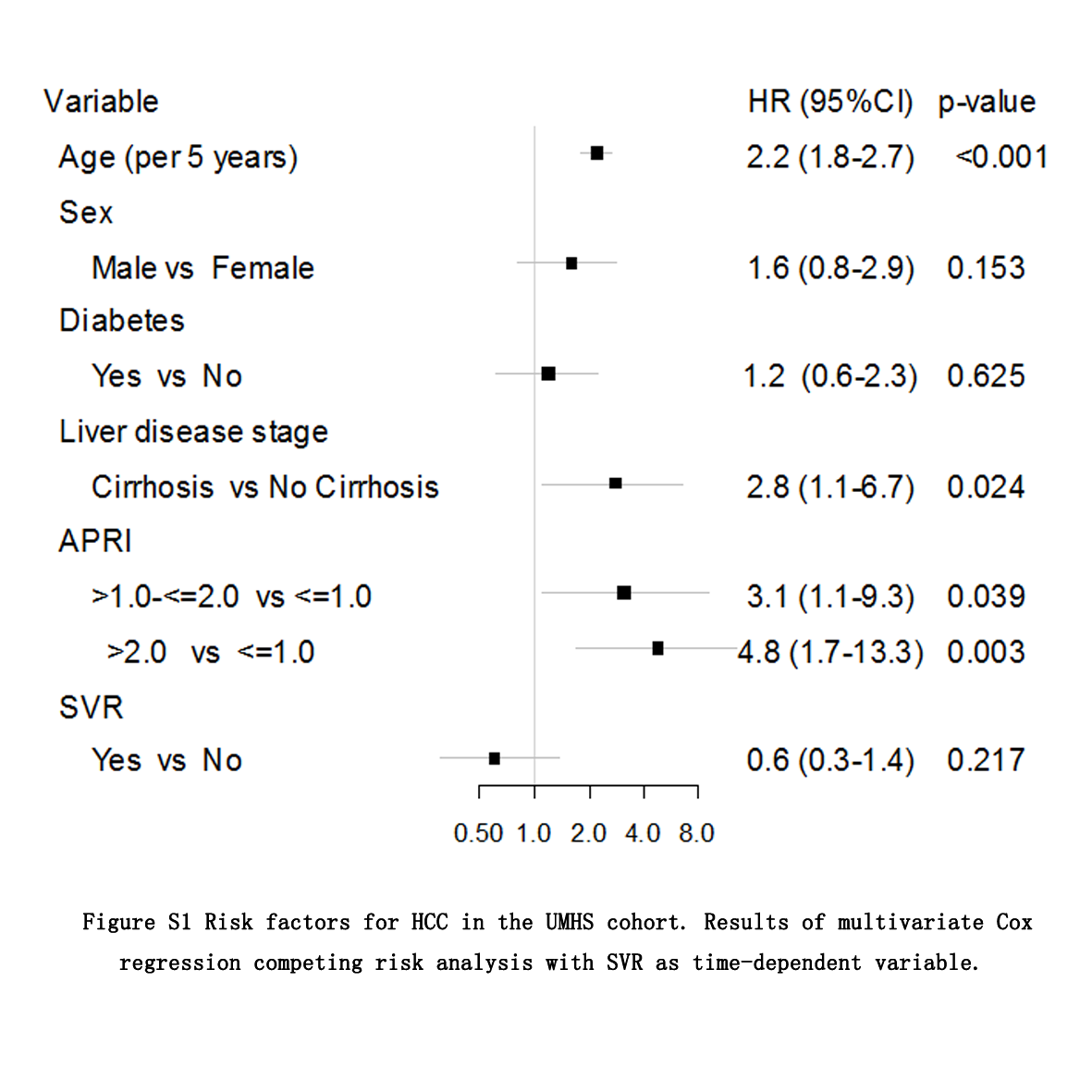

Supplement: Supplementary file 1 — Supplementary Figure 1. [file 41598_2020_77515_MOESM1_ESM.tif]

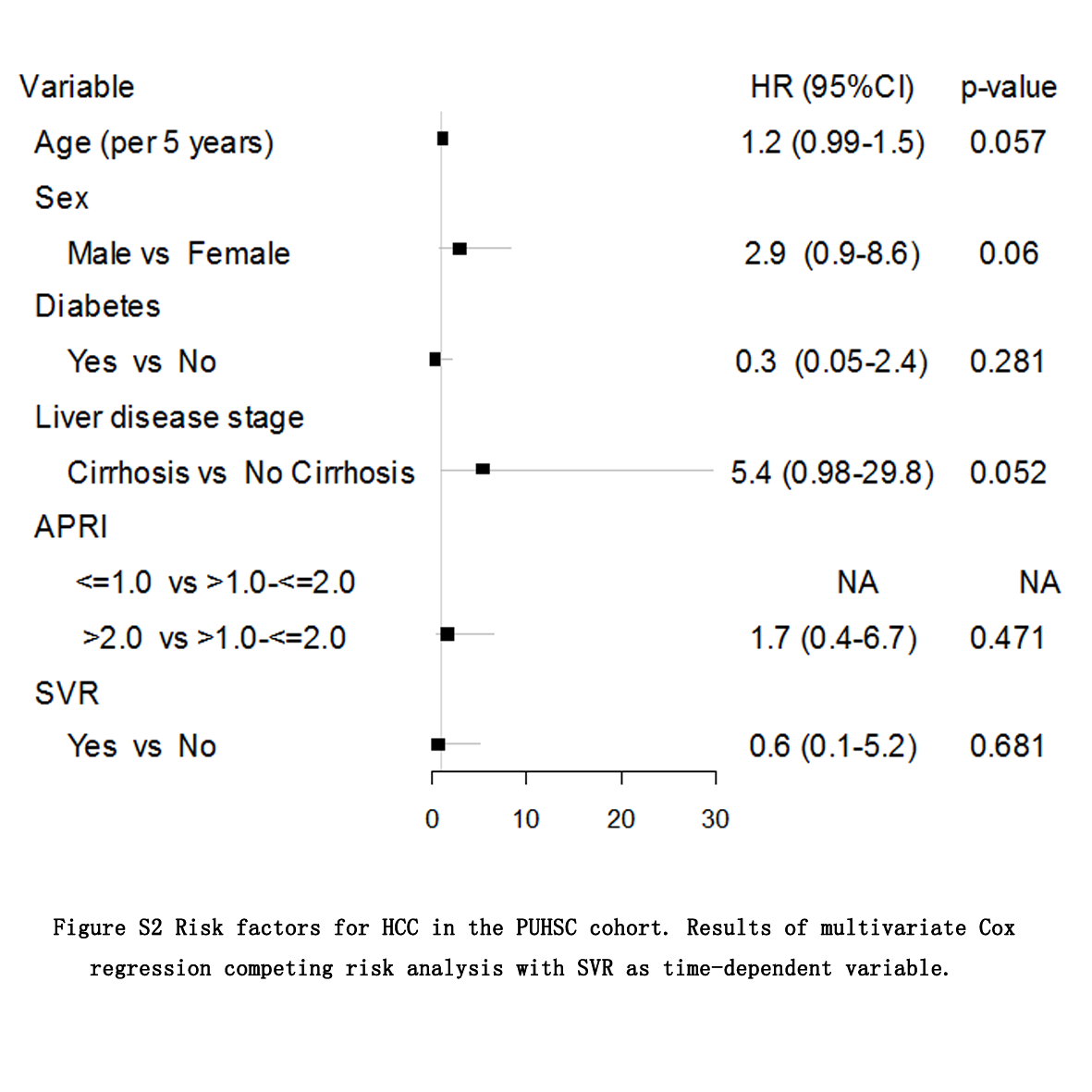

Supplement: Supplementary file 2 — Supplementary Figure 2. [file 41598_2020_77515_MOESM2_ESM.tif]
