# Supplementary material for: Incidence and risk factors of hepatocellular carcinoma in patients with hepatitis C in China and the United States
Source: Sci Rep. 2020 Dec 1;10:20922. doi: 10.1038/s41598-020-77515-y (PMC7708980; doi:10.1038/s41598-020-77515-y)
Supplement: Supplementary file 3 — Supplementary Information 3. [file 41598_2020_77515_MOESM3_ESM.docx]

**Incidence and risk factors of hepatocellular carcinoma in patients with hepatitis C in China and the United States**

Ming Yang^1^, Neehar D. Parikh^2^, Huixin Liu^3^, Elizabeth Wu^2^, Huiying Rao^1^, Bo Feng^1^, Andy Lin^4^, Lai Wei^1^, Anna S. Lok^2^*

^1^Peking University People’s Hospital, Peking University Hepatology Institute, Peking University Health Science Center, Beijing, China.

^2^Department of Internal Medicine, Division of Gastroenterology and Hepatology, University of Michigan, Ann Arbor, MI, United States.

^3^Department of Clinical Epidemiology and Biostatistics, Peking University People’s Hospital, Beijing, China.

^4^The Molecular and Behavioral Neuroscience Institute, University of Michigan, Ann Arbor, MI, United States. * Email: [aslok@med.umich.edu](mailto:aslok@med.umich.edu)

Supplementary Table 1 Incidence of HCC in UMHS and PUHSC cohorts before and after SVR (SVR as a time-fixed variable)

|  | UMHS cohort | | |  | PUHSC cohort | | |  | Total | | |  |  |
| --- | --- | --- | --- | --- | --- | --- | --- | --- | --- | --- | --- | --- | --- |
|  | Patients at risk | Patients developed HCC | HCC incidence (per 100 person-year) |  | Patients at risk | Patients developed HCC | HCC incidence (per 100 person-year) |  | Patients at risk | Patients developed HCC | HCC incidence (per 100 person-year) | HR (95%CI) | *P* value |
| No cirrhosis group | | | | | | | | | | | |  |  |
| No SVR | 140 | 8 | 1.9 (1.0-3.8) |  | 331 | 2 | 0.2 (0.04-0.7) |  | 471 | 10 | 0.6 (0.3-1.2) |  |  |
| SVR | 294 | 0 | NA |  | 385 | 0 | NA |  | 679 | 0 | NA | NA |  |
|  |  |  |  |  |  |  |  |  |  |  |  |  |  |
| Cirrhosis group | | | | | | | | | | | |  |  |
| No SVR | 197 | 30 | 7.0 (4.9-10.0) |  | 79 | 10 | 4.7 (2.5-8.8) |  | 276 | 40 | 6.2 (4.6-8.5) | 1.0 |  |
| SVR | 164 | 7 | 1.0 (0.5-2.1) |  | 59 | 1 | 0.4 (0.06-2.9) |  | 223 | 8 | 0.8 (0.4-1.7) | 0.2 (0.1-0.4) | <0.001 |
|  | | | | | | | | | | | |  |  |
| Overall | | | | | | | | | | | |  |  |
| No SVR | 795 | 38 | 5.0 (3.3-6.2) |  | 854 | 12 | 0.9 (0.5-1.5) |  | 1649 | 50 | 2.2 (1.7-3.0) | 1.0 |  |
| SVR | 458 | 7 | 0.4 (0.2-0.9) |  | 444 | 1 | 0.05 (0.01-0.4) |  | 902 | 8 | 0.2 (0.1-0.4) | 0.1 (0.05-0.2) | <0.001 |

Note: HCC, Hepatocellular carcinoma; SVR, sustained virologic response; HR, hazard ratio; NA, not applicable

Supplementary Table 2 – Univariate competing risk analysis of risk factors for HCC

|  | |  |  | UMHS cohort | |  | PUHSC cohort | |  | Combined cohort | |
| --- | --- | --- | --- | --- | --- | --- | --- | --- | --- | --- | --- |
|  | |  |  | HR (95% CI) | *P* value |  | HR (95% CI) | *P* value |  | HR (95%CI) | *P* value |
| Age (per 5 years) | |  |  | 1.9 (1.6-2.3) | <0.001 |  | 1.4 (1.2-1.6) | <0.001 |  | 1.6 (1.5-1.8) | <0.001 |
| Gender | |  |  |  |  |  |  |  |  |  |  |
|  | | female |  | 1.0 |  |  | 1.0 |  |  | 1.0 |  |
|  | | male |  | 1.6 (0.8-3.0) | 0.15 |  | 2.4 (0.7-7.7) | 0.15 |  | 2.0 (1.1-3.5) | 0.015 |
| BMI | |  |  |  |  |  |  |  |  |  |  |
|  | | underweight and normal weight |  | 1.0 |  |  | 1.0 |  |  | 1.0 |  |
|  | | overweight |  | 1.5 (0.7-3.1) | 0.32 |  | 1.0 (0.3-3.7) | 1.0 |  | 1.6 (0.9-3.1) | 0.13 |
|  | | obese |  | 0.9 (0.4-2.0) | 0.82 |  | 2.0 (0.5-7.4) | 0.30 |  | 1.6 (0.8-3.2) | 0.16 |
| DM | |  |  |  |  |  |  |  |  |  |  |
|  | | no |  | 1.0 |  |  | 1.0 |  |  | 1.0 |  |
|  | | yes |  | 1.5 (0.8-2.9) | 0.21 |  | 0.8 (0.1-6.3) | 0.84 |  | 1.9 (1.1-3.4) | 0.03 |
| Alcohol | |  |  |  |  |  |  |  |  |  |  |
|  | | never |  | 1.0 |  |  | 1.0 |  |  | 1.0 |  |
|  | | current/past consumption |  | 0.8 (0.4-1.5) | 0.51 |  | 2.2 (0.7-6.6) | 0.15 |  | 1.6 (0.9-2.6) | 0.09 |
| Smoking | |  |  |  |  |  |  |  |  |  |  |
|  | | never |  | 1.0 |  |  | 1.0 |  |  | 1.0 |  |
|  | | current/past consumption |  | 1.2 (0.6-2.4) | 0.68 |  | 2.1 (0.7-6.1) | 0.18 |  | 2.4 (1.3-4.3) | 0.003 |
| Coffee | |  |  |  |  |  |  |  |  |  |  |
|  | | never |  | 1.0 |  |  | 1.0 |  |  | 1.0 |  |
|  | | current/past consumption |  | 1.2 (0.6-2.2) | 0.54 |  | 1.7 (0.2-12. 3) | 0.61 |  | 2.6 (1.6-4.4) | 0.0002 |
| HCV genotype | |  |  |  |  |  |  |  |  |  |  |
|  | | non-type I |  | 1.0 |  |  | 1.0 |  |  | 1.0 |  |
|  | | type I |  | 0.9 (0.4-2.0) | 0.82 |  | 4.8 (0.6-38.0) | 0.13 |  | 1.7 (0.8-3.7) | 0.1449 |
| Anti-HBc | |  |  |  |  |  |  |  |  |  |  |
|  | | negative |  | 1.0 |  |  | 1.0 |  |  | 1.0 |  |
|  | | positive |  | 1.1 (0.6-2.0) | 0.85 |  | 1.4 (0.4-4.2) | 0.57 |  | 0.9 (0.5-1.6) | 0.82 |
| PLT (1000/µL) | |  |  |  |  |  |  |  |  |  |  |
|  | | < 100 |  | 1.0 |  |  | 1.0 |  |  | 1.0 |  |
|  | | >= 100 |  | 0.3 (0.2-0.6) | <0.001 |  | 0.1 (0.02 -0.2) | <0.001 |  | 0.2 (0.1-0.3) | <0.001 |
| ALT (per 10 IU/L) | |  |  | 1.01(0.98-1.04) | 0.34 |  | 1.07 (1.04-1.10) | <0.0001 |  | 1.04 (1.02-1.06) | <0.001 |
| AST (per 10 IU/L) | |  |  | 1.06 (1.03-1.08) | <0.001 |  | 1.13 (1.08-1.17) | <0.001 |  | 1.09 (1.07-1.11) | <0.001 |
| ALP (per 10 IU/L) | |  |  | 1.07 (1.04-1.10) | <0.001 |  | 1.10 (1.00-1.19) | 0.04 |  | 1.09 (1.06-1.12) | <0.001 |
| AFP (per 10 ng/mL) | |  |  | 1.04 (0.99-1.10) | 0.12 |  | 1.08 (1.04-1.13) | <0.001 |  | 1.07 (1.03-1.10) | <0.001 |
| ALB (g/dL) | |  |  |  |  |  |  |  |  |  |  |
|  | | < 3.0 |  | 1.0 |  |  | 1.0 |  |  | 1.0 |  |
|  | | >= 3.0 |  | 0.4 (0.2-0.8) | 0.010 |  | 0.1 (0.0-1.1) | 0.07 |  | 0.2 (0.1-0.5) | <0.001 |
| TBIL (mg/dL) | |  |  |  |  |  |  |  |  |  |  |
|  | | < 2.0 |  | 1.0 |  |  | 1.0 |  |  | 1.0 |  |
|  | | >= 2.0 |  | 4.5 (2.4-8.5) | <0.001 |  | 2.4 (0.3-18.6) | 0.40 |  | 5.4(3.0-9.8) | <0.001 |
| INR | |  |  |  |  |  |  |  |  | 1.0 |  |
|  | | < 1.2 |  | 1.0 |  |  | 1.0 |  |  | 1.0 |  |
|  | | >= 1.2 |  | 3.8 (2.0-6.9) | <0.001 |  | 0.00 (0.00-0.00) | <0.001 |  | 4.8 (2.9-8.2) | <0.001 |
| APRI | |  |  |  |  |  |  |  |  |  |  |
|  | | <=1.0 |  | 1.0 |  |  | NA |  |  | 1.0 |  |
|  | | >1.0-<=2.0 |  | 5.2 (1.8-14.8) | 0.002 |  | 1.0 |  |  | 10.2 (3.7-28.1) | <0.001 |
|  | | >2.0 |  | 7.7 (3.0-19.9) | <0.001 |  | 3.9 (1.0-14.4) | 0.045 |  | 20.6 (8.1-52.2) | <0.001 |
| FIB4 | |  |  |  |  |  |  |  |  |  |  |
|  | | <1.45 |  | NA |  |  | NA |  |  | NA |  |
|  | | 1.45-3.25 |  | 1.0 |  |  | 1.0 |  |  | 1.0 |  |
|  | | >3.25 |  | 6.9 (2.5-19.4) | <0.001 |  | 22.0 (2.8-170.5) | 0.003 |  | 11.7 (4.7-29.3) | <0.001 |
| Baseline LSM (kPa) | |  |  | NA |  |  | 1.0 (1.0-1.1) | <0.001 |  | NA |  |
| Liver disease stage | |  |  |  |  |  |  |  |  |  |  |
|  | | no cirrhosis |  | 1.0 | <0.001 |  | 1.0 | <0.001 |  | 1.0 | <0.001 |
|  | | cirrhosis |  | 4.9 (2.3-10.6) |  |  | 30.2 (6.7-137.1) |  |  | 11.0 (5.6-21.8) |  |
| SVR (time-dependent) | | |  |  |  |  |  |  |  |  |  |
|  | not achieved | |  | 1.0 |  |  | 1.0 |  |  | 1.0 |  |
|  | achieved | |  | 0.6 (0.3-1.4) | 0.28 |  | 0.4 (0.1-3.3) | 0.43 |  | 0.8 (0.4-1.6) | 0.53 |
| SVR (time-fixed) | | |  |  |  |  |  |  |  |  |  |
|  | never achieved | |  | 1.0 |  |  | 1.0 |  |  | 1.0 |  |
|  | achieved | |  | 0.1 (0.05-0.3) | <0.001 |  | 0.05 (0.01-0.4) | <0.0001 |  | 0.1 (0.05-0.2) | <0.001 |

Note: HCC, Hepatocellular carcinoma; HR, hazard ratio; BMI, body mass index; DM, diabetes mellitus; HCV, hepatitis C virus; Anti-HBc, antibody to hepatitis B core antigen; PLT, platelet; ALT, alanine aminotransferase; AST, aspartate aminotransferase; ALP, alkaline phosphatase; AFP, alpha fetoprotein; ALB, albumin; Tbil, total bilirubin; INR, international normalized ratio; APRI, aspartate aminotransferase to platelet ratio index; FIB-4, Fibrosis index based on 4 factors; LSM, liver stiffness measurement; SVR, sustained virologic response.
